# From attention to memory along the dorsal-ventral axis of the medial prefrontal cortex: some methodological considerations

**DOI:** 10.3389/fnsys.2014.00160

**Published:** 2014-09-08

**Authors:** Helen J. Cassaday, Andrew J. D. Nelson, Marie A. Pezze

**Affiliations:** ^1^School of Psychology, University of NottinghamNottingham, UK; ^2^School of Psychology, Cardiff UniversityCardiff, UK

**Keywords:** attention, associative leaning, object recognition memory, anterior cingulate, prelimbic, infralimbic, dopamine

## Abstract

Distinctions along the dorsal-ventral axis of medial prefrontal cortex (mPFC), between anterior cingulate (AC), prelimbic (PL), and infralimbic (IL) sub-regions, have been proposed on a variety of neuroanatomical and neurophysiological grounds. Conventional lesion approaches (as well as some electrophysiological studies) have shown that these distinctions relate to function in that a number behavioral dissociations have been demonstrated, particularly using rodent models of attention, learning, and memory. For example, there is evidence to suggest that AC has a relatively greater role in attention, whereas IL is more involved in executive function. However, the well-established methods of behavioral neuroscience have the limitation that neuromodulation is not addressed. The neurotoxin 6-hydroxydopamine has been used to deplete dopamine (DA) in mPFC sub-regions, but these lesions are not selective anatomically and noradrenalin is typically also depleted. Microinfusion of drugs through indwelling cannulae provides an alternative approach, to address the role of neuromodulation and moreover that of specific receptor subtypes within mPFC sub-regions, but the effects of such treatments cannot be assumed to be anatomically restricted either. New methodological approaches to the functional delineation of the role of mPFC in attention, learning and memory will also be considered. Taken in isolation, the conventional lesion methods which have been a first line of approach may suggest that a particular mPFC sub-region is not necessary for a particular aspect of function. However, this does not exclude a neuromodulatory role and more neuropsychopharmacological approaches are needed to explain some of the apparent inconsistencies in the results.

## Introduction

The interconnectivity of medial prefrontal cortex (mPFC) and hippocampus is well-established (Gabbott et al., 2005) and although there are differences between rat and primate mPFC a number of functional homologies have been documented. Indeed data from rat models has confirmed the importance of functional interactions between hippocampus and mPFC (Aggleton et al., 1995; Chudasama et al., 2004; Vertes, 2006; Ragozzino, 2007; Bizon et al., 2012). The signature effect of mPFC lesions is generally viewed as one of cognitive inflexibility (Ragozzino, 2007), in particular when animals need to respond adaptively based on changed reinforcement contingencies (de Bruin et al., 1994; Aggleton et al., 1995; Bussey et al., 1997; Ragozzino et al., 1999; Birrell and Brown, 2000; Killcross and Coutureau, 2003). Such deficits in executive function tend to manifest as perseverative behavior—animals continue to respond according to previously acquired contingencies. Importantly, the delineation of the role of mPFC in executive function has high translational relevance given that mPFC dysfunction results in neuropsychiatric dysfunctions such as schizophrenia (Andreasen et al., 1992; Barch et al., 2001; Spieker et al., 2012).

However, the mPFC is not a uniform entity. Evidence from neuroanatomical studies, including cytoarchitectonics, suggests a distinct heterogeneity to the structure comprising sub-regions such as anterior cingulate cortex (AC) as well as the prelimbic (PL) and infralimbic (IL) cortices. These sub-regions are also distinguishable on the basis of their differing patterns of interconnectivity (Fisk and Wyss, 1999; Heidbreder and Groenewegen, 2003; Vertes, 2004). Thus, hodologically, there are good grounds to expect that the mPFC sub-regions should be dissociable in relation to distinct aspects of cognitive function (Kesner and Churchwell, 2011).

The higher order executive functions which support complex goal-directed behaviors have been an understandable focus of studies of mPFC function (Kesner and Churchwell, 2011; Bissonette et al., 2013). At the same time, there is an appreciation of the need to understand executive functions such as working memory and cognitive flexibility in terms of a “toolbox” of underlying mechanisms (Bizon et al., 2012). The successful dissociation of these mechanisms is likely to require consideration of the functional heterogenity of mPFC as well as the adoption of behavioral tests suitable to tap component processes. Indeed, behavioral dissociations have been demonstrated, particularly with respect to the distinction between AC, PL, and IL, predominantly using tasks which tax attention, learning and/or memory. However, until recently, the majority of studies have employed mechanical, electrolytic or excitotoxic lesions to explore these functional subdivisions (Heidbreder and Groenewegen, 2003). Whilst reinforcing the role of interconnected structures within functional brain circuits, the neuropsychological evidence obtained by studying the effects of lesions which damage fibers and/or cell bodies indiscriminately does not speak to the role of specific neurotransmitter systems in modulating function. Similarly, whilst they provide an important advance to distinguish effects on encoding vs. retrieval, reversible inactivation studies do not specifically address the role of DA function and the border of the temporary lesion is typically ill-defined (Ragozzino, 2007). And, whilst providing important information about functional dissociations between sub-regions with excellent anatomical resolution, the majority of electrophysiological studies do not speak to the role of particular neurotransmitters either.

Evidence from psychopharmacological studies is necessary in order to develop neuropsychopharmacological theories as to the function of mPFC and interconnected brain structures. Dopamine (DA) is a likely neuromodulator of mPFC function in that the region is richly innervated by DA fibers originating in the ventral tegemental area (Lindvall et al., 1974). Consistent with this proposition, the modulation of DA functioning within mPFC has been shown to play an important role in regulating learning and memory, as well as other cognitive functions (Goldman-Rakic, 1998; Robbins, 2000; Robbins and Roberts, 2007).

Microinfusion studies too are not without their limitations. Although they provide a workable method to activate or inactivate particular receptor subtypes within brain regions of interest, their anatomical resolution is less precise and rarely verified. Indeed, studies microinjecting agents selective for different DA receptor subtypes have typically targeted mPFC as an entity rather than attempting any systematic comparison between mPFC sub-regions (Ragozzino, 2002; Floresco et al., 2006b).

For the purposes of the present review, four tasks which have been systematically examined in relation to their dependence on mPFC sub-regions will be considered in some detail, particularly in relation to the available evidence pertaining to the role of DA. The serial reaction time task measures early attentional processing and a key role for mPFC has been established using excitotoxic lesion approaches (Passetti et al., 2002; Pezze et al., 2009). Studies using excitotoxic lesions have also been conducted to examine the role of mPFC in latent inhibition (Lacroix et al., 1998, 2000b; Schiller and Weiner, 2004; George et al., 2010). Earlier studies of trace conditioning used aspiration (Kronforst-Collins and Disterhoft, 1998; Weible et al., 2000) and electrolytic lesions (McLaughlin et al., 2002; Runyan et al., 2004), both of which methods also destroy fibers of passage. However, the role of mPFC has since been confirmed by a variety of other approaches including excitotoxic lesions (Table 1).

**Table 1:** **The behavioral effects associated with changes in neuronal activity in anterior cingulate (AC), prelimbic (PL), and infralimbic (IL) sub-regions of medial prefrontal cortex (mPFC) in tasks measuring serial reaction time, latent inhibition, trace conditioning and object recognition, as well as related benchmark tests of learning and memory (for which the effects of similar interventions in different mPFC sub-regions have been examined)**.

**Behavioral test**	**Lesion, drug, neuronal activity**	**mPFC sub-region**		
		**AC**	**PL**	**IL**
Serial reaction time	Excitotoxic lesion	Reduced accuracy, increased latencies to collect reward, increased omissions (Passetti et al., 2002; note lesion centered on Cg1)	Increased perseverative responding, transient effect on accuracy (Passetti et al., 2002; note lesion included PL and IL)	Increased perseverative responding, transient effect on accuracy (Passetti et al., 2002; note lesion included PL and IL)
		Reduced accuracy and decreased impulsive responding (Chudasama et al., 2003, 2012; note lesion included PL and IL)	Reduced accuracy, slower response latencies, increased omissions and premature responses (Pezze et al., 2009; note lesion included PL and IL)	Increased impulsive responding (Chudasama et al., 2003, 2012)
	Single unit recording	Neuronal responses higher when accurately responding to cue, lower before an incorrect response and increased after an incorrect response (Totah et al., 2009)	Neuronal responses higher when accurately responding to cue, lower before an incorrect response, but no significant change after an incorrect response (Totah et al., 2009)	
	Reversible inactivation		Lower dose muscimol increased premature responding (Paine et al., 2011; note injection coordinates included IL)	Muscimol increased the number of premature responses (Murphy et al., 2012)
			Higher dose muscimol decreased accuracy and impulsive responding (Pezze et al., 2014) and increased omissions (Murphy et al., 2012; Pezze et al., 2014)	
	Disinhibition	SRB95531 decreased accuracy and increased omissions (Pehrson et al., 2013)	Bicuculine decreased accuracy, increased omissions and increased latencies to collect rewards (Paine et al., 2011; note bicuculine likely spread to IL)	Bicuculine blocked the increase in premature responding otherwise caused by CPP (Murphy et al., 2012)
			Picrotoxin decreased accuracy and increased omissions (Pezze et al., 2014)	
	Micro-injection of NMDA antagonist	MK801 increased omissions (Pehrson et al., 2013)		CPP increased the number of premature responses (Murphy et al., 2012)
Reinforced responding in the Skinner box	Excitotoxic lesions	No effect (Risterucci et al., 2003)	Increased premature responding and disrupted the distribution of preparatory responding during the intervals between cues (Risterucci et al., 2003)	Increased premature responding and disrupted the distribution of preparatory responding during the intervals between cues (Risterucci et al., 2003)
Reinforced responding in the Skinner box (Continued)	Single neuron recording		Fast transient responses to sucrose delivery (Burgos-Robles et al., 2013)	Delayed prolonged responses to sucrose delivery (Burgos-Robles et al., 2013)
	Reversible inactivation		Muscimol without effect on collection of earned sucrose rewards (Burgos-Robles et al., 2013)	Muscimol delayed collection of earned sucrose rewards (Burgos-Robles et al., 2013)
Pre-pulse inhibition	Excitotoxic lesion		No effect (Lacroix et al., 1998; note lesion included AC and IL)	No effect (Sullivan and Gratton, 2002; note lesion encroached on PL)
			Increased by larger lesions (Lacroix et al., 2000b; note lesion included AC and IL)	
	Micro-injection of D_1_ antagonist		Impaired (Ellenbroek et al., 1996; note injection coordinates included IL)	Impaired (Shoemaker et al., 2005)
			Impaired (Shoemaker et al., 2005)	
Latent inhibition	Electrolytic lesion	No effect (Joel et al., 1997)	No effect (Joel et al., 1997; note lesion included AC and IL)	No effect (Joel et al., 1997)
	Excitotoxic lesion		No effect (Lacroix et al., 1998, 2000b; Schiller and Weiner, 2004; note lesions centered on PL, encroached on IL and/or AC)	Enhanced (George et al., 2010)
			No effect (George et al., 2010)	
	6-OHDA lesion		Enhanced (Nelson et al., 2010)	No effect (Nelson et al., 2010)
Trace conditioning	Aspiration lesion	Impaired acquisition (Kronforst-Collins and Disterhoft, 1998; Weible et al., 2000)		
	Electrolytic lesion		Impaired acquisition but only at longer CS duration (McLaughlin et al., 2002)	
	Erk inhibition		Impaired retention but not acquisition (Runyan et al., 2004)	
	Single neuron recording	Attentional responses to CSs (Weible et al., 2003; Hattori et al., 2014)	Increased activity to the trace conditioned CS, including within the trace interval (Gilmartin and McEchron, 2005)	Decreased activity to the trace conditioned CS, no change in trace interval activity (Gilmartin and McEchron, 2005)
			Persistent activity within the trace interval during retention tests (Hattori et al., 2014)	
Trace conditioning (Continued)	Reversible inactivation	Impaired acquisition (Kalmbach et al., 2009; note “caudal” placement included PL)		
	Excitotoxic lesion *c-fos* expression	Impaired by lesion and associated with increased neuronal activity in Cg1 but not Cg2 (Han et al., 2003)		
	Excitotoxic lesion	Impaired by immediate-post-training lesions (Oswald et al., 2010)	Impaired by 1-week-post-training lesions (Oswald et al., 2008, 2010)	
Fear conditioning	Microstimulation	No effect on the expression or extinction of conditioned fear (Vidal-Gonzalez et al., 2006)	Increased the expression of conditioned fear and prevented extinction (Vidal-Gonzalez et al., 2006)	Decreased the expression of conditioned fear and facilitated extinction (Vidal-Gonzalez et al., 2006)
	Single neuron recording		Decreased activity during extinction and extinction memory in male rats; females showed increased activity during extinction (Fenton et al., 2014)	Increased activity during extinction and extinction memory (no sex difference; Fenton et al., 2014)
	Reversible inactivation		Muscimol impaired fear expression, but had no effect on extinction memory (Sierra-Mercado et al., 2011)	Muscimol had no effect of fear expression, but impaired extinction memory (Sierra-Mercado et al., 2011)
Object recognition—identity	Excitotoxic lesion	No effect (Ennaceur et al., 1997)	No effect (Ennaceur et al., 1997; Barker et al., 2007)	
	Reversible inactivation		No effect (Hannesson et al., 2004a)	
	6-OHDA lesion		No effect (Nelson et al., 2011a)	No effect (Nelson et al., 2011a)
Object recognition—recency	Excitotoxic lesion		Impaired (Barker et al., 2007; note lesion encroached on IL)	
	Reversible inactivation		Impaired (Hannesson et al., 2004a)	
	6-OHDA lesion		Impaired (Nelson et al., 2011a; note PL lesion anatomically selective)	Impaired (Nelson et al., 2011a; note IL lesion encroached on PL)
	Single neuron recording	Increased activity when in location of a “missing” object at 6 h or 30 day delay (Weible et al., 2012)		
Object recognition—location	Excitotoxic lesion	No effect unless lesion extended to include retrosplenial cortex (Ennaceur et al., 1997)	No effect (Ennaceur et al., 1997; Barker et al., 2007)	
Object recognition—location (Continued)	6-OHDA lesion		No effect (Nelson et al., 2011a)	Impaired (Nelson et al., 2011a)
Spatial recognition memory	Reversible inactivation		No effect on spatial recognition memory, impaired spatial temporal order memory (Hannesson et al., 2004b)	
Spatial memory	Reversible inactivation	Impaired working memory in radial arm maze (Seamans et al., 1995)	Impaired reference memory (Seamans et al., 1995)	
	Micro-injection of D_1_ antagonist		Disrupted performance if 30 min delay between training and test (Seamans et al., 1998)	
	Electrolytic lesions	No effect on non-matching to position (Joel et al., 1997)	Some transient impairment in non-matching to position but more marked impairment in rule reversal (Joel et al., 1997; note lesion extended to AC)	

Both latent inhibition and trace conditioning are simple Pavlovian conditioning procedures in which the associability of the conditioning cue is manipulated, by stimulus pre-exposure in the case of latent inhibition or by the introduction of a time interval after cue offset in the case of trace conditioning. There are a variety of theoretical interpretations of latent inhibition, some of which are attentional, but its psychological mechanisms are still disputed (Lubow and Weiner, 2010). Trace conditioning similarly results in reduced stimulus associability but has been argued to tap working memory because of the need to bridge the time gap introduced by the trace interval (Sweatt, 2004). Similarly, there is good evidence that mPFC is involved in recognition memory as measured by tasks exploiting rats' preferences to explore novelty as presented by previously unseen objects, less recently encountered objects, or familiar objects in novel locations. Some such studies have been driven by the theoretical views on the function of mPFC, some by the known effects of hippocampal lesions on working memory. However, the role of different neuromodulators within these mPFC sub-regions is still poorly understood.

The present review will therefore address the functional distinction between AC, PL, and IL mPFC sub-regions with respect to the mechanisms of attention, learning, and memory. Table 1 summarizes the behavioral effects associated with changes in neuronal activity in AC, PL, and IL mPFC sub-regions in tasks measuring serial reaction time, latent inhibition, trace conditioning and object recognition, as well as some related tests of learning and memory (for which the effects of similar interventions in different mPFC sub-regions have been examined). The advantages and disadvantages of the available neurophamacological techniques—for example in neurotoxic lesion, microinfusion and pharmacogenetic studies—to establish the role of DA will also be considered.

## Early attention: the serial reaction time task

In rodents, the five choice serial reaction time task (5-CSRT) test, which resembles the human continuous performance test, is a commonly used test of attention (Robbins, 2002; Chudasama and Robbins, 2006; Lustig et al., 2013). In this task rodents are trained to detect a brief visual target presented randomly in one out of an array of five apertures in order to obtain food rewards. The proportion of nose pokes into the correct aperture, as opposed to incorrect apertures, provides a measure of attention. Other measures such as correct response latency (for example) may also reflect attentional deficits; whereas magazine latency to collect food reward provides useful information about levels of activity or motivation. Additionally, this task indexes response control or behavioral inhibition, as reflected in the number of premature responses (when the animal responds before the presentation of the light) and perseverative responses (when the animal carries on nose poking in the same aperture after a correct or an incorrect response).

### Excitotoxic mPFC lesions impair 5-CSRT performance

The effects of excitotoxic mPFC lesions have been particularly well-characterized using the 5-CSRT. For example, it has been shown that complete mPFC lesions impair choice accuracy and increase both omissions and perseverative responses, as well as latency to respond correctly (Passetti et al., 2002; Pezze et al., 2009). More discrete lesions have helped to clarify the contribution of specific mPFC sub-regions. Indeed, Passetti et al. (2002) also compared the effects of complete mPFC excitotoxic lesions with those of AC and more ventral PL plus IL mPFC lesions. When more restricted lesions were employed, those with the AC placements resulted in reduced accuracy in the absence of any effect on perseverative responding, although there was some transient increase in the number of omissions (Passetti et al., 2002). In contrast, animals with complete mPFC lesions showed impaired accuracy combined with increased perseverative responding in the absence of any significant change in omissions or latencies (Passetti et al., 2002).

In a later study from the same laboratory, excitotoxic lesions using the same mPFC coordinates as those used by Passetti et al. (2002), reduced accuracy was seen in conjunction with slower response latencies, increased omissions and premature responses (Pezze et al., 2009). It has also been shown that whilst both combined lesions of AC and PL (Chudasama et al., 2003; Chudasama and Robbins, 2006) and lesions restricted to AC (Passetti et al., 2002) impair attentional performance measured as accuracy, lesions restricted to IL increase impulsive responding (Chudasama et al., 2003, 2012).

### Temporary inactivation of mPFC impairs 5-CSRT performance

Complementary to the study of permanent excitotoxic lesions, temporary inactivation studies have also helped to clarify the differential engagement of mPFC sub-regions in attention and response control. Paine and co-workers reported that low doses of muscimol (6.25 to 50 ng/side), a GABA(A) receptor agonist, selectively increased premature responding when infused into the PL plus IL mPFC without affecting accuracy (Paine et al., 2011). In contrast, a high dose of muscimol (500 ng/side) decreased response accuracy and increased the percentage of omissions reflecting an attentional deficit when infused into the PL or IL (Murphy et al., 2012). Increased reward-collection latency however indicated that the high dose may have caused motivational impairments. Lower doses of muscimol (62.5 to 250 ng/side) in this case injected into PL have since been found to decrease attentional performance without causing nonspecific impairments in reward-collection latency, consistent with the importance of PL mPFC in attention (Pezze et al., 2014). Moreover, also consistent with the results of the excitotoxic lesion studies reported above, infusion of the N-methyl-D-aspartate (NMDA) receptor antagonist MK801 into the AC selectively impaired attentional parameters of performance (Pehrson et al., 2013). Further confirming the role of IL mPFC in impulsivity, both the NMDA receptor antagonist CPP and muscimol increased the number of premature responses when infused into the IL (Murphy et al., 2012).

### Disinhibition in mPFC impairs 5-CSRT performance

Disinhibition of mPFC may also cause selective attentional deficits. When infused into the PL/IL, the GABA(A) receptor antagonist bicuculine (25 ng/side) impaired attentional performance and induced motivational disturbances (Paine et al., 2011). Similarly infusion of picrotoxin, also a GABA(A) receptor antagonist, into PL mPFC results in attentional deficits in the absence of non-specific impairments (Pezze et al., 2014). Yet another GABA(A) receptor antagonist SRB95531 also selectively reduced attentional aspects of performance after AC infusion (Pehrson et al., 2013). On the other hand when infused in IL the GABA(A) receptor antagonist bicuculine blocked the increase in premature responding otherwise caused by the NMDA receptor antagonist CPP (Murphy et al., 2012). These data converge in suggesting that while the more dorsal parts of the mPFC (AC plus PL) may drive attentional functions, the IL region rather controls inhibitory behavior.

### Electrophysiological correlates of 5-CSRT performance

This dissociation based on attentional vs. inhibitory behaviors has been further confirmed by electrophysiological studies of the differential involvement of mPFC sub-regions, in this case in the ability to allocate preparatory attention to improve subsequent stimulus processing and response selection. Totah and co-workers have used single unit recording in a three choice serial reaction time task to show that levels of activity in both PL and AC are related to preparatory attention in the period before the presentation of the light cue (Totah et al., 2009). In this study, neuronal responses were higher when the rat was accurately responding to the cue, lower before an incorrect response and not different from baseline before an omission. The authors suggested that both PL and AC may be involved in signaling reward expectation of the upcoming goal-relevant stimuli. However, in contrast to the neural activity in PL, neural activity in the AC increased after an incorrect response, suggesting that this structure also codes for error related events.

### DA modulates 5-CSRT performance

Consistent with the importance of DA as a modulator of early attentional processes, a history of amphetamine exposure also appears to alter performance on the 5-CSRT test. For example, rats which have been trained to self-administer amphetamine showed a transient deficit in attention reflected by decreased accuracy and increased omissions when tested on the 5-CSRT (Dalley et al., 2005). More recently, it has been reported that repeated and intermittent exposure to escalating doses of amphetamine in rats increased the number of omissions during the amphetamine treatment or the withdrawal phase (Fletcher et al., 2007). When the attentional load of the task was increased by shortening the stimulus duration, the level of accurate responding was also decreased in amphetamine pre-treated rats. The similarity of the impairments to those caused by lesion of the dorsal mPFC suggested that the effect of amphetamine treatment on attention may have been mediated by the dorsal mPFC.

A few more anatomically specific neurochemical studies have investigated the role of DA release in the mPFC in attention and/or response control (Robbins, 2002). Using post-mortem neurochemistry a positive correlation between 3,4-dihydroxyphenylacetic acid (DOPAC)/DA ratio and choice accuracy was observed in the right hemisphere (Puumala and Sirvio, 1998). In freely moving rats, *in vivo* microdialysis has confirmed increased extracellular levels of DA in the PL and IL sub-regions of animals performing a simplified one choice version of the task (Dalley et al., 2002b). In the same study, it was demonstrated that increased DA levels in the mPFC of rats treated with an amphetamine challenge paralleled increased numbers of premature responses; and post-mortem neurochemistry revealed that impulsive rats had a higher level of DA turnover in both PL and IL sub-regions of the mPFC (Dalley et al., 2002a). Thus, these neurochemical studies point to the conclusion that whilst attention clearly requires DA in the mPFC, too much may trigger impulsive responding.

While profound catecholamine depletions of the mPFC did not alter baseline performance on the 5-CSRT, deficits emerged when the rats were behaviorally challenged. For example, the introduction of a variable inter-trial-interval (ITI) to make the target stimulus less predictable induced a deficit in accuracy in rats with 6-OHDA lesion of the mPFC (Robbins et al., 1998; Robbins, 2002). Moreover, microinfusion studies have confirmed the importance of DA D_1_ receptor transmission, in attentional processes in particular (Dalley et al., 2004; Boulougouris and Tsaltas, 2008). Granon and co-workers showed an enhancement in attentional processes after infusion into PL mPFC of the DA D_1_ receptor agonist SKF38393, though only at low levels of attentional performance (Granon et al., 2000). This effect was subsequently replicated using a more selective D_1_ receptor agonist SKF81297, in a variation of the task in which rats were trained to detect a visual target and then to remember the location of this visual stimulus (Chudasama and Robbins, 2004). Moreover, the decline in accurate responding and the increase in omissions induced by amphetamine withdrawal could be prevented by infusion of the D_1_ receptor agonist SKF38393 into the PL mPFC (Fletcher et al., 2007). In contrast, the D_1_ receptor antagonist SCH23390 impaired the accuracy of attentional performance in rats that performed at high baseline levels (Granon et al., 2000). This set of data may reflect an inverted U-shaped function relating attentional processes to DA-receptor stimulation in the mPFC. Similarly, working memory (Sawaguchi and Goldman-Rakic, 1991; Williams and Goldman-Rakic, 1995; Goldman-Rakic, 1995; Zahrt et al., 1997) and the expression of conditioned fear (Pezze et al., 2003) have also been suggested to require an optimal level of prefrontal dopamine-receptor stimulation.

### 5-CSRT conclusions

**Figure 1:**
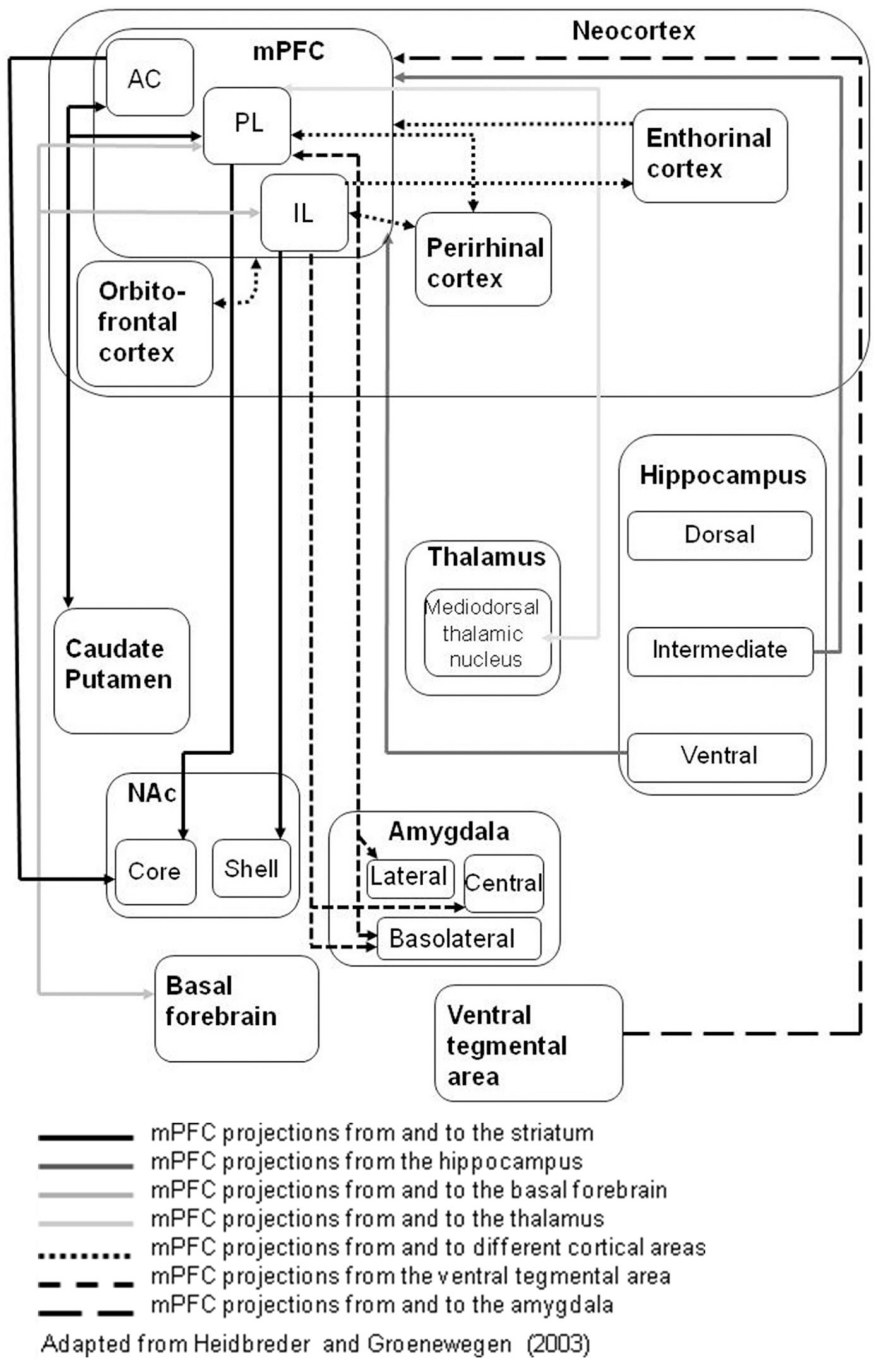
**The connections of rat medial prefrontal cortex (mPFC) sub-regions with other key brain regions; based on Heidbreder and Groenewegen (2003)**. The different arrow line formats (use of dashed and gray lines) denote connections to (and from) different target structures.

Thus, neurochemical evidence converges to suggest that mPFC DA is involved in early attentional processes, as well as in response inhibition (see *Executive Functions in mPFC* below). In common with other functions mediated in mPFC, as well as the more established interconnectivity with hippocampus (Gabbott et al., 2005), there is evidence pointing to the importance of its interconnections with other brain regions such as NAc (Pezze et al., 2007, 2009). For example, the attentional dysfunction demonstrated in the serial reaction time task after mPFC lesions was ameliorated by treatment with the DA D_2_ antagonist sulpiride administered intra-NAc (Pezze et al., 2009). This finding points to the importance of neuromodulation, specifically by DA D_2_ receptors within NAc. However, to date these studies have not distinguished PL vs. IL mPFC sub-regions, nor the core vs. shell NAc sub-regions with which they are differentially interconnected (Figure 1).

Effective early attention or vigilance of the kind measured by the 5-CSRT is necessary but not sufficient for the efficient functioning of subsequent learning and memory processes. For example, early attention is a likely modulator of associability or animals' capacity to learn about environmental cues. However, this subsequent capacity is not directly assessed in the 5-CSRT.

## Attentional learning: latent inhibition

Effects on associability or later learning are measured in latent inhibition procedures. Latent inhibition is the reduction in later learning which will normally result from pre-exposure to the designated conditioning cue (conditioned stimulus, CS) without any initial consequences, at least in the first instance (Lubow and Moore, 1959). The reduction in later learning is demonstrated when the pre-exposed CS subsequently comes to predict an outcome (unconditioned stimulus, US). In attentional terms, such a pre-exposed cue can be seen to be irrelevant. Consistent with a likely role for mPFC in latent inhibition, NAc and other interconnected regions such as entorhinal cortex, hippocampus, orbitofrontal cortex and basolateral amygdala (BLA), are known to modulate the expression of latent inhibition (Honey and Good, 1993; Tai et al., 1995; Coutureau et al., 1999; Schiller and Weiner, 2004; Gal et al., 2005). Consequently, a number of studies have been conducted to examine the role of mPFC in latent inhibition.

### Excitotoxic mPFC lesions can enhance latent inhibition

Contrary to expectation, a number of studies examining the effects of electrolytic lesions to the mPFC and its sub-regions as well as excitotoxic lesions (typically spanning two to three mPFC sub-regions) reported no effect on latent inhibition (Joel et al., 1997; Lacroix et al., 1998, 2000b; Schiller and Weiner, 2004). More restricted lesions have also been reported to be without effect (Joel et al., 1997).

However, a number of neuromodulatory effects are known to depend on the strength of the behavioral effect under examination in that ceiling or floor effects can preclude the demonstration of enhanced or impaired performance, respectively. This has certainly proven to be an important consideration in studies of latent inhibition. For example, whilst abolition of latent inhibition is most readily demonstrated when the effect is clear in the control group, the demonstration of potentiated latent inhibition may require a weakened level of latent inhibition in the control group. Weakened latent inhibition is produced experimentally either by using a low number of stimulus pre-exposures or a high number of conditioning trials (Weiner and Arad, 2009). Indeed, such parametric manipulations have been found to be critical to the demonstration of the effects of lesions in BLA or core NAc and which do not disrupt latent inhibition under conditions which reliably produce latent inhibition in control (in this case sham-operated) animals, but which enhance latent inhibition under behavioral conditions known to be insufficient to produce latent inhibition in controls (Gal et al., 2005; Schiller and Weiner, 2005). In one previous study which similarly examined the effects of lesions to mPFC under conditions which did not produce latent inhibition in controls (in this case using a high number of conditioning trials), still no effects of mPFC lesions were demonstrated (Schiller and Weiner, 2004).

The functional heterogeneity of mPFC also needs to be taken into account and lesions restricted to IL mPFC have more recently been reported to potentiate latent inhibition (George et al., 2010). This study used 30 stimulus pre-exposures in a within-subject aversive on-the-baseline procedure, under which experimental conditions enhanced latent inhibition was demonstrated in a group with excitotoxic lesions restricted to IL mPFC but not PL mPFC. Since the demonstration of the potentiating effects of IL mPFC lesions did not in this case require the use of any experimental manipulation to weaken latent inhibition, the on-the-baseline procedure used by George et al may be particularly sensitive to small variations in the magnitude of latent inhibition. The previous study which reported no effect of IL lesions used the standard off-the-baseline procedure (Joel et al., 1997).

There has been little further investigation—using experimental parameters that do result in latent inhibition in untreated animals—of the involvement of the PL and IL mPFC sub-regions in relation to the expression of latent inhibition. However, with respect to sub-region there is evidence that the critical IL vs. PL lesion site to produce this effect depends on whether the lesion is made excitotoxically (Schiller and Weiner, 2004; George et al., 2010) or using more neurochemically selective neurotoxins (Nelson et al., 2010).

### DA and latent inhibition

The abolition of latent inhibition after treatment with systemic amphetamine has been demonstrated many times (Weiner et al., 1981, 1984, 1988; Weiner, 1990, 2003; Weiner and Arad, 2009) and this effect of amphetamine has been one of the key arguments used to support the use of latent inhibition as a behavioral marker for schizophrenia (Weiner, 1990, 2003; Weiner and Arad, 2009). Unsurprisingly therefore, further studies have gone on to examine the role of mPFC DA. When 6-OHDA was used to lesion DA terminals within PL vs. IL mPFC, it was the injection in PL which lead to the emergence of latent inhibition under weak pre-exposure conditions insufficient to produce latent inhibition in controls (Nelson et al., 2010). These results are contrary to those reported with excitotoxic lesions made with ibotenic acid, in that in this case the effective lesion was the ventral placement corresponding with IL mPFC (George et al., 2010). The fact that opposing patterns of outcome have been demonstrated depending on whether the lesion method has greater or lesser neurochemical selectivity clearly points to the importance of neuromodulation by DA within PL vs. IL mPFC. This would be consistent with other evidence that DA within mPFC modulates cognitive control (Goldman-Rakic, 1998; Robbins, 2000; Robbins and Roberts, 2007). However, 6-OHDA lesions are not selective to DA and typically also deplete noradrenalin (NA; see also *Methodological Considerations* below).

On balance the available evidence point to the importance of mPFC sub-region, selectivity of the lesioning method to catecholamines and the level of latent inhibition generated by the behavioral parameters in use. For example, the results obtained with 6-OHDA IL lesions, which in fact depleted DA in both the IL and PL and were without effect on latent inhibition (Nelson et al., 2010), can be seen as consistent with the earlier reported finding that larger electrolytic or excitotoxic mPFC lesions do not affect latent inhibition (Joel et al., 1997; Lacroix et al., 1998, 2000b; Schiller and Weiner, 2004). Such dissociable effects of anatomically complete vs. partial mPFC lesions mirror the observation that total (shell plus core) and sub-regionally-selective (shell vs. core) NAc lesions have opposing effects on latent inhibition (Gal et al., 2005).

Further direct evidence on the neuromodulatory role of DA is lacking in that within mPFC local infusions of either D_1_ or D_2_ antagonists (Ellenbroek et al., 1996) as well as treatment with the indirect agonists amphetamine and apomorphine (Lacroix et al., 2000a) have been found to be without effect on latent inhibition. However, it must be noted that these microinfusion studies were conducted using standard latent inhibition procedures more suited to test for the abolition of latent inhibition rather than any potential enhancement; neither did they involve any comparison of effects in relation to PL vs. IL placement. Thus, given the importance of both the precise behavioral parameters in use and the placement with respect to sub-region, although consistently negative to date, the available evidence to date suggests that the role of DA within PL may yet be confirmed by microinfusion studies.

### Latent inhibition conclusions

Overall, the evidence base for the role of DA in mPFC sub-regions is more limited in the case of latent inhibition compared with that available in the case of the 5-CSRT. A particular role for DA in more dorsal mPFC regions would be broadly consistent with the importance of AC and PL sub-regions in 5-CSRT accuracy as distinct from impulsive responding (*Early Attention: the Serial Reaction Time Task*; Table 1). However, to our knowledge there has to date been only one study to examine the role of AC in latent inhibition and this was an electrolytic lesion study. The latent inhibition procedure nonetheless adds to our understanding in that it allows for examination of the effects of attention on associative learning. However, the assessment of such effects is inevitably indirect and potentially confounded by other cognitive demands introduced by the latent inhibition task, such as the need to switch flexibly between stimulus-reinforcement contingencies. The interface between attention and executive function is discussed below (*Executive Functions in mPFC*).

## Working memory: trace conditioning

Trace conditioning is the reduction in learning produced by the introduction of a trace interval between CS offset and US onset. Like latent inhibition, trace conditioning can be demonstrated in a variety of Pavlovian conditioning procedures. Also in common with latent inhibition, attentional processes have been identified as essential to the normal acquisition of trace conditioning. However, unlike latent inhibition, trace conditioning has been argued to represent an essential aspect of working memory. The ability to bridge time delays to show associative learning in a trace conditioning procedure allows animals to associate what goes with what, when potentially causally-related events are separated in time. With respect to anatomical substrates it is very well-established that conventional hippocampal lesions impair trace conditioning, and this effect is reproducible in a variety of conditioning procedures (though typically aversively motivated: McEchron et al., 2000; Beylin et al., 2001; Quinn et al., 2002).

Consistent with known projections from hippocampus, mPFC has also been shown to be part of the trace conditioning network. Following a number of lesion studies of eye blink conditioning in the rabbit which showed the importance of mPFC sub-regions including the caudal aspect of AC (Kronforst-Collins and Disterhoft, 1998; Weible et al., 2000) single neuron recording studies indicated an attentional role for AC, manifest particularly in the early acquisition of trace conditioning (Weible et al., 2003; Oswald et al., 2008; Hattori et al., 2014). A similar effect, in this case of electrolytic lesions to PL mPFC in the same eye blink conditioning preparation, has also been reported; however—consistent with some functional difference—the demonstration of PL lesion effects on acquisition was found to require the use of a relatively long duration CS (McLaughlin et al., 2002).

### Excitotoxic mPFC lesion effects on trace conditioning

Further studies using the eye blink conditioning preparation have been conducted with more anatomically selective excitotoxic lesions. For example, the effects of post-training PL lesions point to the importance of PL in regulating retrieval: PL lesions made 1 week after training resulted in reduced expression of prior learning, however animals showed normal relearning (Oswald et al., 2008, 2010). Again using the same behavioral procedures, reduced expression of prior learning was found to result from excitotoxic AC lesions but in this case only if the lesions were made immediately post-training (Oswald et al., 2010). This dissociation between the effects of PL and AC lesions based on the timing of the lesions with respect to behavioral training points to a functional difference in relation to the retrieval of consolidated material in PL vs. processes related to consolidation which require AC (Oswald et al., 2010). Since initial acquisition was not examined in the Oswald et al. studies (only relearning), the importance of AC for early attentional processing in trace conditioning is not excluded.

### Convergent evidence for dissociations by mPFC sub-region

Consistent with the above studies by Oswald et al. (2008, 2010), in a rat CER procedure, inhibition of extracellular signal-regulated (Erk) cascade to disrupt long term memory storage within PL impaired the retention but *not* the acquisition of trace conditioning (Runyan et al., 2004). Thus, there is convergent evidence consistent with the importance of PL in retrieval-related processes. Moreover, excitoxic AC lesions have been reported to reduce trace conditioning in a rat CER procedure in the same way as an experimental distractor stimulus (based on the putative attentional role of AC: Han et al., 2003).

Comparing across a variety of trace conditioning preparations, the emerging pattern seems to be a role for (1) AC in early attentional processing of the CS and/or acquisition (Kronforst-Collins and Disterhoft, 1998; Weible et al., 2000, 2003; Han et al., 2003; Kalmbach et al., 2009; Hattori et al., 2014); and (2) PL when memory processes are more directly engaged, for example when retention is tested (Runyan et al., 2004; Oswald et al., 2008, 2010), when neuronal activity is examined during a relatively long trace interval (Gilmartin and McEchron, 2005) or when longer CS durations compound the memory load (McLaughlin et al., 2002). This pattern is broadly consistent with that emerging from the 5-CSRT which also points to a particular role of AC in attentional rather than later stages of processing. In the case of trace conditioning, interventions restricted to IL have been little examined to date. Although the available data are as yet limited, there is some indication of further functional differentiation with still more ventral placements in IL, in the form of differences in electrophysiological profile in a study which directly compared IL with PL neuronal responses. This functional differentiation took the form of increased vs. decreased activity in PL vs. IL mPFC during conditioning with a 20 s trace interval (Gilmartin and McEchron, 2005).

### DA and trace conditioning

With respect to the role of DA, systemic amphetamines increased conditioning over a 30 s trace interval measured in a CER procedure (Norman and Cassaday, 2003; Horsley and Cassaday, 2007). The amphetamines are indirect catecholamine agonists so these results suggest a role for DA or NA in the neuromodulation of trace conditioning. However, particularly in the case of trace conditioning, an additional consideration arises in that lesion effects have typically been investigated in aversively motivated procedures (eye blink or CER). Lesions and drug treatments affecting the DA system have been found to have different effects in aversive (Norman and Cassaday, 2003; Horsley and Cassaday, 2007) and appetitive trace conditioning procedures (Kantini et al., 2004; Cassaday et al., 2005). This difference may relate to the different motivational systems engaged by aversive and appetitive conditioning procedures or inevitable differences in the salience of the aversive and appetitive UCSs used in such studies. Consistent with the latter interpretation, systematic comparison of the effects of mPFC lesions in two aversive trace conditioning variants, motivated by relatively mild corneal air puff vs. relatively aversive peri-orbital eye shock UCS showed greater trace conditioning deficits with the air puff UCS (Oswald et al., 2010). Therefore, it will be important to establish the role of mPFC DA using appetitive as well as aversive task variants, particularly given the evidence that the relationship between mPFC DA and cognition follows an inverted U-function (Zahrt et al., 1997; Arnsten, 1998).

Reduced trace conditioning can be viewed as a cognitive impairment. Conversely, enhanced trace conditioning can be taken to reflect cognitive enhancement because of improved working memory function (Sweatt, 2004), as demonstrated after both treatment with amphetamines (Norman and Cassaday, 2003; Horsley and Cassaday, 2007) and catecholaminergic depletion within NAc (Nelson et al., 2011d). Connections of the AC (caudal aspect) point to the importance also of the cholinergic system at least in the attentional processes necessary to trace conditioning (Weible et al., 2007). However, the role of other key neuromodulators within mPFC and the extent to which its sub-regions are differentially involved in trace conditioning are not yet established. A role for mPFC DA is to be expected, and the interconnectivity with NAc is consistent with the pattern of effects observed in latent inhibition procedures (Nelson et al., 2010, 2011b,c). Indeed, we have presented some preliminary evidence for differential effects of PL and IL dopamine depletion on trace conditioning measured in a CER procedure (Thur et al., 2010). However, statistically this result was marginal and needs replication. DA depletion in core rather than shell NAc provided the most likely explanation of the increased trace conditioning demonstrated in our earlier study (Nelson et al., 2011d). Accordingly, based on the known interconnectivity between NAc core and shell with PL and IL mPFC, respectively, this effect would be predicted to be reproduced by DA depletion in PL. We have recently found that the early acquisition of appetitive trace conditioning is modulated by the DA D_1_ agonist SKF81297 but we have so far been unable to distinguish the role of different mPFC sub-regions (Pezze et al., unpublished data).

### Trace conditioning conclusions

In summary, there is some strong evidence for the functional significance of mPFC sub-regions from studies of trace conditioning, including some intriguing parallels with conclusions emerging from studies with the 5-CSRT. Together with evidence from systemic drug studies this points to the role of DA within mPFC as a likely modulator of trace conditioning also. However, these lines of enquiry have not yet led to any compelling neuropsychopharmacological studies of trace conditioning.

## What, where, and when: object recognition memory

Recognition memory relies on the ability to discriminate familiar “known” stimuli from novel stimuli which have not previously been encountered, or which have been seen in a different location or in the same location but less recently. The fact that rats have a natural tendency to preferentially explore relatively novel cues, provided by the intrinsic features of objects (“what”), new spatial context (“where”), or mere forgetting over time (“when”), has been exploited to devise tests of object recognition which do not require any additional motivation or training. Such familiarity-based discrimination tests have been widely used to investigate the neurobiological basis of recognition memory (Ennaceur and Delacour, 1988; Dere et al., 2007; Winters et al., 2008; Warburton and Brown, 2010).

As in the case of serial reaction time processing, latent inhibition and trace conditioning, structures interconnected with hippocampus have been identified as key. As might be expected, hippocampus is essential for recognition discriminations based on the spatial location of items, the where aspect of familiarity (Wan et al., 1999; Hardt et al., 2009). Perirhinal cortex has been found to be necessary for both object recognition (Aggleton et al., 1997; Bussey et al., 1999; Brown and Aggleton, 2001) and the processing of temporal information, to allow the performance of familiarity discriminations based on the timing or recency of the encounter (Barker et al., 2007). Thus, perirhinal cortex plays an important role in mediating both what and when aspects of familiarity. There is also good evidence that the mPFC sub-regions modulate different aspects of recognition memory.

### Electrophysiological studies of object recognition

Electrophysiological studies have shown that mPFC neurons respond selectively to particular objects (Rainer and Miller, 2000, 2002; Xiang and Brown, 2004). One recent study neatly distinguishes electrophysiological responses related to memory for objects as distinct from any unconditioned responses directly elicited by the objects themselves. Specifically, neurons in AC increased activity when mice returned to the location of a “missing” object (that was previously explored at that location and had since been removed). This increased activity was seen in response to the absent object at both 6 h and 30 day delay and was therefore argued to reflect memory, given the delays in use—most likely consolidation-related processes—as distinct from activity related to object exploration (Weible et al., 2012). Whilst an elegant method to distinguish unconditioned effects, this experimental design cannot dissociate memory for features inextricably related to object identity from memory for object location. However, some lesion studies of mPFC allow comparison across different object recognition variants.

### Inactivation and lesion studies of object recognition

Specifically, although mPFC does not seem to be necessary for object recognition as measured by the standard test variant based on object identity (Ennaceur et al., 1997; Hannesson et al., 2004a), performance on object recency variants has been shown to be disrupted by either reversible inactivation of Hannesson et al. (2004a) or excitotoxic lesions to Barker et al. (2007) PL mPFC. Thus, the available evidence suggests that although—to the extent that mPFC lesions are without effect in standard tests of object recognition—mPFC may not be necessary for familiarity judgments *per se*, it nonetheless plays an important role in familiarity based on when an object has been presented. The role of mPFC in familiarity based on where an object has been presented is less well-established. Performance in object location variants has been reported to be disrupted by excitotoxic lesions to AC plus retrosplenial cortex. However, excitotoxic lesions restricted to AC were without effect under the same test conditions and neither was there any effect of relatively selective PL lesions on object location recognition (Ennaceur et al., 1997).

### DA and object recognition

To delineate the role of neuromodulators such as DA within mPFC requires the use of neuropharmacological approaches. For example, the effects of 6-OHDA lesions to the PL and IL mPFC sub-regions have been systematically compared across novel object recognition variants requiring judgments about object identity, recency, and location. Consistent with earlier reports that mPFC is not involved in simple novel object recognition based on intrinsic features of the object, DA depletion in PL/IL left performance based on object identity intact. In contrast, in the recency variant of the task, lesions to both PL and IL interfered with animals' ability to differentiate between earlier presented (“old”) and more recently presented (“familiar”) objects (Nelson et al., 2011a). These findings point to a role of mPFC DA in familiarity based on when an object has been presented.

With respect to the neuroanatomical locus of neuromodulation by DA within mPFC, the lesion study with 6-OHDA points to a role for PL DA (and/or NA). The PL lesion which was sufficient to impair object recognition based on recency, was anatomically highly selective and spared catecholamine content in the more ventral IL (Nelson et al., 2011a). This selectivity of the behaviorally effective PL lesion in turn suggests that the same behavioral deficit seen also in the IL-lesioned group was due to catecholamine loss produced by the lesion in the PL rather than the IL or entire mPFC (Nelson et al., 2011a). The importance of PL mPFC is consistent with neuroanatomical data. There are reciprocal interconnections between the perirhinal cortex and the mPFC, as well as behavioral evidence to suggest that functional interactions between these regions mediate recognition memory performance requiring recency judgments (Hannesson et al., 2004a; Barker et al., 2007). Notably, connections are particularly strong between perirhinal cortex and PL rather than IL mPFC (Vertes, 2004; Hoover and Vertes, 2007).

Neurotoxic 6-OHDA lesions which depleted DA in IL mPFC also partially disrupted recognition memory for object location; however in the case of the location variant DA depletion in PL was without effect (Nelson et al., 2011a). The 6-OHDA IL lesions in use significantly depleted catecholamines also in the PL, but since at the same time animals with selective PL lesions performed at comparable levels to shams, the impairment in object recognition based on location was attributed to IL (Nelson et al., 2011a). Thus, the results of this study suggest that DA within IL may contribute to the ability to detect a change in the spatial location of a familiar object. Previous studies which showed intact object location memory used excitoxic lesions rather than DA depletion, were targeted at the PL or entire mPFC rather than the IL and were conducted using the basic variant of object displacement (Ennaceur et al., 1997; Barker et al., 2007).

### Underlying memory mechanisms

A variety of further object location recognition variants have been developed, some of which introduce contextual associations (by introducing new distinctive environments) in order to manipulate familiarity in relation to the test environment (Dere et al., 2007). One further aspect of context is the spatial relations between the objects themselves: in object-in-place variants, for example, of an array of four objects two swap positions (Dix and Aggleton, 1999). These contextual variants are likely to tap different underlying memory mechanisms. Indeed, excitotoxic mPFC lesions and inactivation with lidocaine are known to depend on the contextual vs. spatial object recognition variant in use in that they can disrupt memory for object-in-place whilst leaving discriminations based on object location intact (Ennaceur et al., 1997; Hannesson et al., 2004a; Barker et al., 2007). In other words, the effects of mPFC lesions depend on whether successful discrimination requires recognition of the topographical relationship between sets of objects in a particular context or the detection of some alteration in the spatial location of a familiar object. To our knowledge the effects of DA depletion or treatment with DA agonists have not been examined in the object-in-place task variant.

With respect to those variants for which the role of mPFC has been examined, performance based on when the objects were presented finds its simplest account in the assumption that rats will preferentially explore the object seen the longest time ago because the memory trace for that object will be weaker than the memory trace of the object sampled most recently; in other words, rats discriminate on the basis of relative recency rather than on the basis of the exact order of occurrence of the objects (Ennaceur, 2010). However, it remains possible that performance could depend on more precise temporal order memory in that successful discrimination would also result if animals remembered the exact order in which they were previously exposed to the objects (Mitchell and Laiacona, 1998; Hannesson et al., 2004a; Barker et al., 2007; Barker and Warburton, 2011). This distinction matters in relation to how theories of mPFC account for its role in “when” processing. There is also independent evidence to suggest that mPFC lesions produce deficits on tasks that require the integration of temporal information (Kesner and Holbrook, 1987; Kesner, 1989) and such findings are consistent with the hypothesis that the temporal sequencing of behavior is a key aspect of mPFC function (Fuster, 2001; Dalley et al., 2004; see also *Temporal Control of Responding* below). Notwithstanding these other data, it is difficult in effect to differentiate between the two alternative accounts of performance on the recency task empirically (Ennaceur, 2010; Barker and Warburton, 2011). Moreover, impairments on the recency task manifest behaviorally as equivalent exploration of two objects presented at the test stage of the procedure. Therefore, lesion effects could arise because both objects were identified as equally novel or because both were recognized as equally familiar (Ennaceur, 2010).

### Object recognition conclusions

On balance, the evidence from object recognition lesion (including 6-OHDA and reversible inactivation) studies points to a particular role for mPFC DA (Nelson et al., 2011a) in the regulation of aspects of recognition memory, particularly if the task conflates the need to make recency judgments (Hannesson et al., 2004b) or spatial aspects of contextual processing (Ennaceur et al., 1997; Hannesson et al., 2004a; Barker et al., 2007).

However, a full consideration of the role of mPFC DA requires an additional level of analysis to examine the role of the different receptor subtypes. Although lesion methods suggest that mPFC is not necessary for object recognition as measured by the standard test variant based on object identity (Ennaceur et al., 1997; Hannesson et al., 2004a; Nelson et al., 2011a), this does not exclude a modulatory role for mPFC. Indeed, evidence that the role of DA D_1_-like receptors in the consolidation of novel object recognition memory (de Lima et al., 2011) is mediated in mPFC has been provided by evidence for DA D_1_ regulation of protein synthesis after novel object exposure (Nagai et al., 2007) as well by micro-injection experiments comparing the effects of DA D_1_–like receptor agents with DA D_2_ receptor agents in mPFC (Nagai et al., 2007; Rossato et al., 2013). Similarly, we have recently found that novel object recognition is impaired by the DA D_1_ agonist SKF81297, both after systemic injections and after microinfusion directly into PL mPFC (Pezze et al., unpublished data).

With respect to the wider circuitry, the IL mPFC receives input from the hippocampus (Hoover and Vertes, 2007) and hippocampus is similarly necessary for object recognition discriminations based on the spatial location (Wan et al., 1999; Hardt et al., 2009). Moreover, the IL projects to NAc shell (Berendse et al., 1992) and NAc shell DA similarly plays a critical role in object location memory (Nelson et al., 2010). Thus, object location familiarity is likely to depend on a circuit of which IL mPFC is just one component.

Similarly within the general domain of recognition memory, “what, where, and when” components have been particularly attributed to perirhinal cortex, hippocampus and mPFC, respectively. However, these interconnected areas are functionally interdependent so functional dissociations are not absolute (Figure 1). For example, perirhinal cortex plays a role in processing when as well as what has been presented (Hannesson et al., 2004a; Barker et al., 2007); mPFC plays a role in identifying where as well as when an object was presented, in this case with some evidence for dissociation by sub-region (Nelson et al., 2011a).

## Related benchmark tests of learning and memory

The literature on mPFC function is vast and we have primarily focused on four behavioral tasks for which there has been some research effort to distinguish the relative roles of mPFC sub-regions and for which there is evidence for neuromodulation by DA. We should also consider the extent to which related benchmark tests of learning and memory (for which the effects of similar interventions in different mPFC sub-regions have been examined) may rely on the same underlying psychological processes.

### Motivated responding

Excitotoxic lesions to mPFC have been reported to disrupt preparatory responding in a reaction time task requiring lever manipulation, in this case release, in response to presentation of a cue light. IL and PL lesions were compared but in this study there was no difference between these more restricted lesions and whole mPFC lesions, only lesions restricted to AC were without effect. All lesions involving IL and/or PL increased premature responding and disrupted the distribution of preparatory responding during the intervals between the cues (Risterucci et al., 2003). Electrophysiological and pharmacological evidence has recently come from a multichannel single unit recording study which systematically compared the responses of neurons in IL and PL sub-regions whilst rats were lever pressing for sucrose pellets on a variable interval schedule. Whilst a similar proportion of neurons were activated during reinforced lever pressing, the time course of responses in IL and PL was clearly different, as was the response to pharmacological inactivation with muscimol (Burgos-Robles et al., 2013). Lever pressing is a very simple operant response which nonetheless requires attention, learning and memory, as well as motivation, for optimal performance at different stages of training. The rats tested in the above studies were pre-trained and so the findings do not address such distinctions in relation to the component psychological processes. Importantly, 5-CSRT test procedures include a number of parameters suitable to distinguish motivational from attentional effects on accuracy. Motivational impairments can be distinguished as increased latencies to collect food reward and increased omissions.

### Pre-pulse inhibition

As was the case for latent inhibition, initial studies of prepulse inhibition reported no effect of excitotoxic mPFC lesions, either non-selective with respect to sub-region (Lacroix et al., 1998) or targeted to the IL sub-region (Sullivan and Gratton, 2002). However, larger excitotoxic mPFC lesions have since been shown to increase pre-pulse inhibition (Lacroix et al., 2000b). Conversely, prepulse inhibition is dose-dependently reduced by micro-injection of DA D_1_ or DA D_2_ antagonists in mPFC (Ellenbroek et al., 1996). Moreover, the impairment following injection of a D_1_ antagonist can be reproduced by micro-injections targeted to PL or IL (Shoemaker et al., 2005). However, as was the case for the 5-CSRT, an optimal level of prefrontal dopamine-receptor stimulation may be key and infusions of the indirect DA agonist apomorphine in mPFC have also been found to disrupt prepulse inhibition (Lacroix et al., 2000a). Effects of interventions restricted to AC have yet to be examined.

### Fear conditioning

Fear conditioning is an important and influential area of research in its own right and largely beyond the scope of the current review. However, it must be acknowledged that the majority of the latent inhibition and trace conditioning studies conducted to examine the role of mPFC sub-regions have been conducted in CER procedures (Han et al., 2003; Schiller and Weiner, 2004; Nelson et al., 2010) or other aversively motivated procedures such as eyeblink conditioning (Kronforst-Collins and Disterhoft, 1998; Weible et al., 2000). Naturally such studies are routinely run with control groups so that effects on basic conditioning can be distinguished from those specific to latent inhibition or trace conditioning.

Micro-injection studies have identified differential modulation of fear conditioning by mPFC DA D_4_receptors vs. D_1_-like receptors). For example, DA D_4_ activation by PD168077 increased conditioning to low (0.4 mA) intensity foot shock UCS but blocked conditioning to higher intensity (0.8 mA) foot shock UCS when injected in the PL region of mPFC. This finding combined with the fact that there was no effect on the recall of previously learned emotionally salient memories is consistent with a role for DA D_4_ receptors in the encoding of nonsalient (but not salient) aversive outcomes (Lauzon et al., 2009).

There is also evidence for distinct roles of mPFC sub-regions in different aspects of fear conditioning. One highly influential study used reversible inactivation (produced by injecting muscimol) to identify dissociable roles of IL vs. PL in the extinction vs. the expression of conditioned fear (Sierra-Mercado et al., 2011). Moreover, this proposed distinction is consistent with electrophysiological recording and microstimulation studies which have identified a role for PL in the expression vs. the extinction of conditioned fear responses (Vidal-Gonzalez et al., 2006) albeit there is also evidence for sex differences (Fenton et al., 2014). A role of mPFC in the initial acquisition as well as the expression of fear has also been identified, particularly when the task demands for contextual or temporal processing are increased, for example by the introduction of a trace interval (Gilmartin et al., 2014).

### Spatial recognition memory

Rats' tendency to explore novel object locations has been exploited to devise analogous tests of recognition memory in the radial arm maze (Hannesson et al., 2004b). Hannesson et al. (2004b) conducted an analogous series of experiments in the radial arm maze. This study similarly relied on rats' tendency to spontaneously explore but in this case to measure spatial recognition memory (measured as the tendency to explore a novel arm not previously visited) and spatial temporal order memory (measured as preference for the least recently visited arm, based on the order in which arms have been visited). Lidocaine inactivation of mPFC (with the injection targeted in dorsal PL) had no effect on the basic recognition memory variant but impaired task performance based on recency (Hannesson et al., 2004b).

Similarly, although PL and IL placements were not systematically compared, both 6-OHDA lesions to PL mPFC (Bubser and Schmidt, 1990) and infusions of DA D_1_ antagonists (Seamans et al., 1998) have been shown to disrupt spatial memory, as measured in T-maze delayed alternation or in radial arm maze tasks with a delay between training and testing, respectively.

### Spatial working and reference memory

In more standard radial arm maze tests, reversible inactivation produced using lidocaine impaired working memory when targeted in AC whereas impaired reference memory resulted from inactivation targeted in PL (Seamans et al., 1995). A role for DA was established in a later study from the same laboratory in that performance was impaired by micro-injection of a DA D_1_ antagonist targeted on PL mPFC provided there was a delay between training and test (Seamans et al., 1998).

Similarly, delayed-non-matching to sample (using lever position in the Skinner box) has been reported to show some impairment after PL plus AC mPFC lesions. Delayed-non-matching to sample is also a classic test of working memory. However, reduced performance was transient (in that depressed accuracy improved after the introduction of each delay) and was moreover not shown to be delay-dependent and—in the absence of any effect on initial acquisition—rule reversal learning from non-matching to matching variants of the task showed a more marked impairment (Joel et al., 1997). There was no effect of lesions restricted to AC in this study. Thus, the PL may be the critical site for rule shifting in this task, reflecting impairment in executive function rather than working memory (Joel et al., 1997).

Although a few studies have shown little or no spatial deficits after mPFC lesions (Kesner et al., 1989; Granon et al., 1996), on balance it is well-established that the mPFC (Kolb et al., 1983; Aggleton et al., 1995) and in particular its DA innervation is important for aspects of spatial memory (Brozoski et al., 1979; Bubser and Schmidt, 1990; Ragozzino et al., 1998; Seamans et al., 1998).

### Temporal control of responding

Last but not least, the ability to time responses to environmental contingencies is critical to performance in the 5-CSRT as well as other reaction time tasks, and in the various delayed response tasks used to assess working memory in object recognition as well as other standard procedures. Moreover, impaired temporal control can also be expected to disrupt the expression of learning in the Pavlovian procedures considered in this review.

This is likely to be an important confound in that depletion of mPFC DA projections (using 6-OHDA injected into the ventral tegmental area) has been found to impair the temporal control of behavior even in simple reaction time tasks (Parker et al., 2013a). Pharmacological blockade of D1 but not D2 receptors also disrupted the development of anticipatory responses (Parker et al., 2013a). Similarly, interfering with DA function (using viral vectors to block tyrosine hydroxylase, or pharmacological blockade of mPFC D1) impaired the temporal control of behavior in a fixed interval timing task (Narayanan et al., 2012). Moreover selective stimulation of mPFC D1 receptors using optogenetic methods confirmed the pharmacological and anatomical specificity of this finding (Narayanan et al., 2012). Electrophysiological studies from the same laboratory—using a time-estimation task in which a cue was presented on 50% trials—have identified phase locking as a mechanism by which mPFC networks synchronize brain activity to control response times (Narayanan et al., 2013). Thus, there is compelling evidence that DA signaling in mPFC modulates the temporal control of responding which underlies performance across the range of tasks considered here. Evidence suggesting that timing may be a mPFC-mediated aspect of executive function has been fully reviewed elsewhere, and in relation to the cognitive symptoms of Parkinson's disease which include timing deficits (Parker et al., 2013b).

## Executive functions in mPFC

As discussed above, aspects of executive function such as timing may be a key factor across the spectrum of tasks examined. Even attention is not a unitary construct and subsumes vigilance as measured by the serial reaction time task as well as attentional learning in so far as this is measured by latent inhibition procedures. In any event, neither latent inhibition task nor serial reaction time procedures provide pure measures of attentional processes (Weiner, 1990, 2003; Muir, 1996; Lubow and Weiner, 2010). For example, a role for mPFC DA in mediating latent inhibition enhancement through failure to switch attentional resources to the now relevant previously pre-exposed stimulus (Weiner, 1990, 2003) would be consistent with a wider role in integrating information about different stimulus-reinforcement contingencies and adjusting behavior in relation to past vs. present contingencies (Kehagia et al., 2010; Nelson et al., 2010).

Serial reaction time task performance has received almost as much prominence as a potential test of impulsivity or executive function (Chudasama et al., 2003, 2012). Overall, the available data from serial reaction time studies suggest that while the PL and AC control attentional processes, IL mPFC is more involved in generating response inhibition (i.e., in impulsivity or executive function). There is also independent evidence that DA signaling in PL mPFC modulates attentional functions. Interestingly, attentional processes appear to require an optimal level of prelimbic neural activity (Pezze et al., 2014) and DA D_1_ receptor stimulation (Granon et al., 2000). It is known that DA D_1_ receptor stimulation increases the excitability of inhibitory interneurons (Goldman-Rakic, 1998; Seamans et al., 2001; Tseng and O'Donnell, 2007; Trantham-Davidson et al., 2008). Also the appropriate level of neuronal activation known to underlie attentional processes may depend of DA D_1_ receptor stimulation in the PL.

Of course the majority of behavioral tasks involve basic aspects of attention, learning, and memory as well as the higher order executive functions necessary to show behavioral flexibility. Accordingly experimental interventions affecting the DA system may do so at a number of levels and executive function may be a common underlying factor. For example, both NA deafferentation (McGaughy et al., 2008) and direct blockade of DA D_1_ receptors (Ragozzino, 2002) within the mPFC have been shown to disrupt rule shifting; conversely, DA activity in mPFC is increased during reversal learning (van der Meulen et al., 2007).

## Methodological considerations

All methods have their limitations. Most important for present purposes is the trade-off between neuroanatomical and neurochemical resolution which is a feature of the approaches most commonly adopted to date. Excitotoxic lesion and electrophysiological methods offer excellent neuroanatomical resolution but tell us little about the role specifically of DA within mPFC. However, the standard method to deplete DA using 6-OHDA makes lesions which may be less anatomically circumscribed than those made by standard excitotoxic methods. However, this difference may be more apparent than real in that neurochemical lesions are more typically verified by HPLC rather than methods which allow visualization of the boundaries of the lesion. Quantitative immunocytochemistry can be used to determine the final boundaries of such lesions (using for example antibodies against the rate limiting biosynthetic enzymes). However, delineation of lesion boundaries is known to depend on the method in use. For example, excitotoxic lesions show different boundaries using an immunohistochemical marker compared with those identified using conventional Nissl staining for histological assessment (Jongen-Relo and Feldon, 2002). Moreover, HPLC remains the method of choice to quantify changes in other neurotransmitters as well as secondary changes in neurotransmitter function in interconnected brain regions. Functional recovery might be seen to be a particular issue in the case of neurotoxic lesions made with 6-OHDA but the delineation of all lesions is confounded by secondary compensatory changes in response to the damage.

### Selectivity of 6-OHDA lesions

A further constraint on the selectivity of 6-OHDA lesions arises in that the pre-treatments routinely used to protect NA neurons are not reliably successful and changes in NA as well as DA typically result from 6-OHDA lesions in mPFC (Nelson et al., 2010, 2011a) as well as in other brain regions such as NAc (Nelson et al., 2011b,c,d). However, neurotoxic 6-OHDA lesions do not result in non-specific neuronal damage in that they do not—for example—result in significant changes in 5-HT levels in target regions such as mPFC (Nelson et al., 2010, 2011a). Primary and secondary effects of neurotoxic lesions should also be distinguished, but 6-OHDA lesions do not necessarily result in secondary changes in control regions selected on the basis of their known connectivity with the mPFC (Sesack et al., 1989; Takagishi and Chiba, 1991).

Anatomically, the dominant view is that 6-OHDA lesions are typically less well-circumscribed. Infusion of 6-OHDA into the PL can nonetheless produce selective depletion within this target structure and spares catecholamine content in the more ventral IL cortex (Nelson et al., 2010, 2011a). However, infusion of 6-OHDA into the IL has a less selective effect in that it depletes catecholamines in both the IL and more dorsally in the PL (Naneix et al., 2009; Nelson et al., 2010, 2011a). On the other hand, in contrast to the evidence that mPFC lesions can enhance the responsiveness of DA neurons in NAc (Deutch et al., 1990), neither PL nor IL 6-OHDA lesions have any effect on striatal DA (Rosin et al., 1992; Nelson et al., 2010, 2011a). Thus, these lesions are nonetheless in some respects more selective.

### Dose-related effects of drugs administered by microinfusion

Microinfusion studies are needed to identify the role of different receptor subtypes in neuromodulation within mPFC. DA D_2_ receptor family mediated inhibition (Greengard, 2001; Traynor and Neubig, 2005) might be expected to mimic the effects of DA depletion. However, DA has been reported to have excitatory effects within mPFC, mediated via actions at the DA D_1_ receptor family (Gonzalez-Islas and Hablitz, 2003). Thus, the effects of DA depletion may rather be reproduced by DA D_2_ agonists.

Drugs administered by microinfusions into any target structure are likely to spread beyond any intended target sub-region to encroach on adjacent sub-regions (AC vs. PL vs. IL in the case of mPFC). Where a drug spreads by diffusion there will necessarily be a concentration gradient from the tip of the injection cannula extending to all other points in the brain. What matters is whether there will be a high enough concentration within the required radius of influence and at the same time a low enough concentration outside that radius of influence such that adjacent structures function normally.

Since the minimal pharmacologically active concentrations of the drugs of interest are typically unknown, and because in any case there is no practical way of measuring concentration gradients from the point of infusion, the best criterion of spread has to be functional. In other words, where behavioral differences between injections targeted on different sub-regions are demonstrated, these must relate to dose-related effects. Functional spread will inevitably vary depending on the particular receptor agents in use and properties such as molecular weight and lipophilicity. In addition to behavioral measures, there are a variety of other methods to estimate spread of drug after microinjection based on functional criteria. Moveable recording electrodes adjacent to infusion sites have, for example, been used to test the limits of the drug's effectiveness (Edeline et al., 2002). Radiolabelled ligands have also been injected to estimate the radius around the injection site that may be affected by a particular infusion volume. Where a variety of receptor agents are in use, the conclusions drawn are extrapolated to other compounds (Granon et al., 2000).

However, in every case, the precise extent of spread determined will depend not only on the properties of the particular compound injected but also the method to determine the extent of its effectiveness. Logic dictates that at further points of diffusion and lower concentrations the compound will be below threshold for the psychobiological effects of interest. In practice, where differential effects are demonstrated between the two regions, then the functional spread cannot be greater than about 1 mm in diameter (because if it were, one would expect to see the same effects following infusions in both regions). This level of resolution is consistent with the successful demonstration of dose-related effects in shell vs. core NAc (Johnson et al., 1996; de Bruin et al., 2001; McFarland and Kalivas, 2001; Anderson et al., 2003; Floresco et al., 2006a) based on the injection volume (0.3 μ l/side) as established by an earlier *in vitro* autoradiography study with [^3^H]dopamine (Johnson et al., 1996).

Autoradiography can also be used to assess diffusion over time. For example, Granon et al. (2000) injected 0.5 μ l volumes of radiolabelled DA antagonist (in this case in rat prefrontal cortex) and killed animals after 5 or 60 min. As might be expected the area of diffusion was smaller at 5 min than at 60 min but the concentration was greater at 5 min than at 60 min after micro-injection. Thus, spatial vs. temporal resolution is inevitably confounded by techniques to visualize the spread of drug injections within target regions. The functional spread of substances administered by micro-infusion is not routinely verified by autoradiography (presumably because of the inherent limitations of this technique discussed above). However, there is good evidence to suggest that to target treatments specifically to dorsal PL is likely to be a reasonably selective experimental strategy (Hannesson et al., 2004a,b). It has been independently established that the spread of lidocaine can be described by the equation *R* = (3 × *V*/4 × π)^1/3^, in which R represents the effective radius and *V* the volume injected (Tehovnik and Sommer, 1997). On this basis, lidocaine micro-infusions can be assumed to effectively inactivate neurons within in a radius of 0.8–1.3 mm from the infusion site. Therefore, the functional spread of lidocaine injected in PL is likely to result in substantial inactivation of PL with much more modest effects in AC and dorsal IL (Hannesson et al., 2004a,b).

The functional spread of small volumes of other compounds used in micro-infusion studies can similarly be assumed to be of the order of 1 mm, albeit particular receptor agents will inevitably spread differently, in relation to their molecular weight and lipophilicity, for example, as well as any neighboring fiber tracts. And it must be noted that in another respect the anatomical specificity of lidocaine is limited in that it does not discriminate between neurons and fibers of passage. Additionally because compounds injected tend to disperse up the cannula tracts, dorsal regions are more likely to be affected than areas ventral to the initial placement. So, for example, injections targeted on IL encroach on PL under conditions in which the same treatment targeted on PL can have more circumscribed effects (Nelson et al., 2010, 2011a). Similarly, injections in PL are more likely to have unwanted effects in AC which is situated dorsal to the injection site than in IL which is ventral.

For practical reasons, studies with a number of behavioral conditions rarely compare the effects of more than two concentrations of drug. However, an inverted U-function best describes the relationship between DA function and cognitive performance. In other words both under—and over-activation of DA receptors can be sufficient to result in cognitive impairment (Zahrt et al., 1997; Arnsten, 1998). In line with the Yerkes-Dodson law relating arousal to performance, DA functioning in the mid-range optimizes most aspects of behavioral or cognitive performance (Yerkes and Dodson, 1908). Accordingly treatments for disorders involving the DA system are tailored to restore function to the mid-range: Parkinson's disease results from DA depletion and is treated with D_1_ agonists (Zahrt et al., 1997); conversely, chronic stress results in D_1_ overactivity which impairs working memory, and this impairment can be treated with D_1_ antagonists (Zahrt et al., 1997). Appreciation of the inverted U-function relating performance to the level of DA function in mPFC results in some—at face value—counterintuitive predictions. Thus, the importance of a particular receptor sub-type might be established by the demonstration of impairments in behavioral performance following treatments with appropriate doses of DA D_1_ agonists as well as antagonists (Granon et al., 2000; Pezze et al., 2014) As discussed above, DA D_2_ receptor family mediated inhibition (Greengard, 2001; Traynor and Neubig, 2005) means that DA D_1_ agonists might be expected to mimic the effects DA depletion in some brain regions. However, similar arguments apply with respect to the need to keep DA function within the optimal range.

## New methods of working

*In vivo* voltammetry can be used to monitor DA function in awake behaving animals (Stevenson and Gratton, 2003; Champagne et al., 2004). This technique has better temporal and spatial resolution than for example microdialysis and has already been adapted for use with some behavioral tests. Similarly fast-scan cyclic votammetry can be used to measure changes in catecholoamine concentrations in discrete brain regions and with sub-second resolution. The pharmacological resolution is still less than perfect in that fast-scan cyclic votammetry does not necessarily distinguish between DA and NA (Park et al., 2010). However, DA and NA can be distinguished by targeting different microelectrodes to their respective pathways (Park et al., 2011). Fast cyclic voltammetry can also be used to measure the effects of stimulated DA release (O'Connor and Lowry, 2012) and has recently been further refined by the development of an integrated wireless system which allows both recoding and stimulation in awake, freely moving rats (Li et al., 2013). However, although this represents an important technological advance and rats adapt well to the implants necessary for voltammetry studies, less invasive procedures are of course preferable.

Pharmacogenetic approaches which could in principle be applied to delineate the role of DA within mPFC have also developed rapidly over the past decade. First, DA can be modulated indirectly, for example by catechol-o-methyltransferase (COMT) or nicotinamide-N-methyltransferase (NNMT) albeit these enzymes are widely distributed within the brain. Polymorphisms of the COMT gene which might modulate the effects of dopaminergic drugs have been suggested to provide one mechanism on which more specific genotype-based neuropharmacological agents might act selectively to modulate DA, in this case with some regional specificity to PFC, and leaving subcortical DA systems unaffected (Apud and Weinberger, 2006). Moreover, multiple COMT mRNA variants have been identified in human brain, including human PFC, and it is likely that these alternate gene products are of functional importance (Tunbridge et al., 2007), for example in relation to cognitive ability (Starr et al., 2007). COMT plays a role in the normal degradation of DA. However, this effect is seen throughout the brain and regionally selective effects have yet to be demonstrated. More extreme effects are associated with NNMT, an enzyme in a metabolic pathway via which dopaminergic toxins may be produced in the brain. NNMT is known to occur naturally in the human brain and thus may contribute to idiopathic neurodegenerative disease processes. For example, NNMT mRNA levels have been found to be reduced post-mortem frontal cortex taken from schizophrenic patients (Bromberg et al., 2012).

Micro-RNA molecules target hundreds of genes and are of great interest in the identification of biomarkers for disease states (Hunsberger et al., 2009). For example, consistent with the disruption of systems and networks rather than dysfunction in any single neurotransmitter system such as DA, post-mortem gene expression studies also point to the importance of differences in microRNA in the brains of schizophrenic patients (see Chana et al., 2013, for review). Conversely, specific DA receptors have also been directly implicated, for example in a study comparing gene expression in schizophrenia and bipolar disorder (Zhan et al., 2011). However, medication can itself result in significant changes, for example in mPFC DA D1 receptor density (Knable et al., 1996). Studies with knockout mice in which a particular DA receptor is congenitally absent can also be used to identify the functional roles of particular receptor subtypes. For the most part such studies have provided evidence consistent with that obtained from systemic drug studies using adult animals. For example, in the case of latent inhibition there is complementary evidence for the role of DA D_1_ as well as D_2_ receptors (Bay-Richter et al., 2009, 2013; Nelson et al., 2012). Thus, pharmacogenetic approaches have been useful to provide convergent evidence with respect to key DA receptor subtypes. However, gene expression is inactivated throughout the brain and from the earliest stages of development in standard knockout models. Thus, although a useful first line of approach, they do not allow us to identify the key underlying pathways that would allow the development of still better targeted pharmacogenetic tools.

The full delineation of mPFC function requires both neuroanatomical and neuropharmacological resolution. Where the key pathways have been identified, by lesion or micro-infusion studies of the kind reviewed above, rapidly developing technologies support still more targeted pharmacogenetic interventions. Inducible knockouts or conditional deletions in which the target gene can be inactivated at a specific time and in specific tissues allow a more fine-tuned approach. Moreover, the role of synaptic plasticity in particular brain regions can be investigated using viral vectors to deliver microRNA, for example in perirhinal cortex to investigate its role in object recognition (Scott et al., 2012). Indeed, for a number of years, viral vectors have been used to induce receptor over-expression in particular target regions: for example of 5-HT_1b_ in NAc shell resulting in sensitization to the effects of cocaine (Neumaier et al., 2002) or increased alcohol consumption (Hoplight et al., 2006); or of DA D_1_ in PL mPFC resulting in increased consumption of sweet solutions as well as stronger associations between addictive drugs and contextual cues (Sonntag et al., 2014). Whilst to date addiction research has been a particular focus of such studies, the wider potential application of localized pharmacogenetic interventions is clear (Hommel et al., 2003).

The technology to produce localized interventions is progressing rapidly. Viral vectors are relatively safe and have low toxicity and at the same time provide for highly effective transduction of genetic material into target cells. Nanodevices are nonetheless the preferred approach to develop regionally-selective interventions which may in the longer term prove suitable for clinical trials. Already nanotechnology has been used to provide vectors carrying silencing RNA in order to selectively interfere with gene expression in particular dopamine signaling pathways; the expression of key proteins can be reduced in the absence of any signs of cytotoxicity (Bonoiu et al., 2009). As discussed above, convergent evidence including the use of viral vectors and optogenetic methods confirmed the role of mPFC D1 receptors in the temporal control of behavior, thereby identifying a key circuit which could serve as a target for the treatment of dopaminergic diseases (Narayanan et al., 2012). Thus, new methodological approaches to the functional delineation of the role of mPFC in attention, learning and memory have great potential. In the longer terms epigenetic effects of drug treatments may also be identified with particular mPFC sub-regions and their wider networks (Csoka and Szyf, 2009).

## Conclusions and implications

A variety of cognitive tasks—including measures of executive function—rely on attention, learning and memory and in this sense any quest for “pure” tests may be misguided (Muir, 1996; Bizon et al., 2012). Nonetheless behavioral distinctions based on task reveal some consistency in the data (Table 1). One emerging pattern is that mPFC is important for the integration of information across the attentional (5-CSRT, latent inhibition), temporal (trace conditioning) and contextual demands of the task (also for the what, where, when components of object recognition). With respect to mPFC sub-regions, there is evidence to suggest that AC has a relatively greater role in attention, whereas IL is more involved in executive function. Arguably, the data are as much consistent with a gradation of specialization along its dorsal-ventral axis as clear functional segregation between AC, PL, and IL sub-regions. However, the final answer on this point rests on the resolution of the methods adopted to date. Moreover, the conventional lesion methods which have been a first line of approach because of their excellent anatomical resolution do not exclude a neuromodulatory role and more neuropsychopharmacological approaches are needed to explain some of the apparent inconsistencies in the results.

Already a number of findings point to the importance of DA within mPFC sub-regions—in the in the attentional processing required for successful performance in the 5-CSRT, in attentional learning as measured by latent inhibition, as well as in different aspects of object-recognition memory. We have also considered some evidence pertaining to wider networks of interconnected brain areas such as hippocampus and NAc, as well as other cortical areas, in the case of object recognition for example (Figure 1). Over and above established dose-related effects, it is also increasingly apparent that within any particular mPFC sub-region, perturbations—whether caused by permanent damage, reversible inactivation methods or drug microinfusions—with at face value opposing neuropharmacological effects, can result in equivalent behavioral outcomes. For example, both inactivation and disinhibition of the PL mPFC caused attentional deficits in spite of opposite effects on mPFC neural activity, as demonstrated using *in vivo* electrophysiology (Pezze et al., 2014). Thus, as has already been suggested for working memory (Goldman-Rakic, 1995; Rao et al., 2000), attention too seems to require an optimal level of neuronal activation of the PL mPFC, with too little or too much neuronal activity being detrimental.

**Figure 2:**
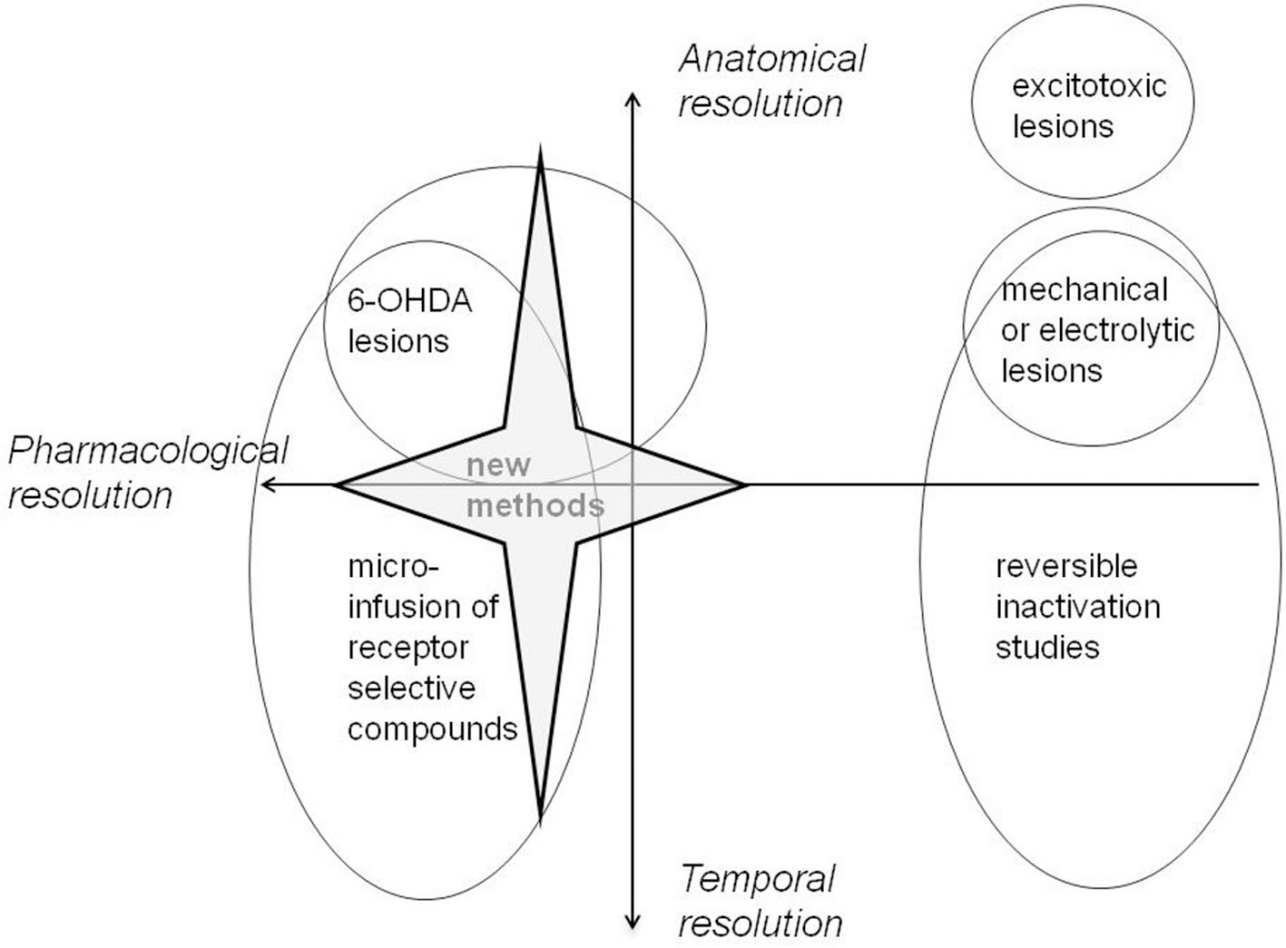
**A schematic representation of the anatomical (how precisely located), temporal (how well-specified in terms of duration of action), and pharmacological resolution afforded by the techniques most widely available to study medial prefrontal function *in vivo***. Whilst there is generally a trade-off between anatomical and temporal resolution, this schematic representation breaks down for some of the newer neuropsychopharmacological methods. For example, fast-cyclic voltammetry has fantastic temporal resolution combined with excellent anatomical resolution. Pharmacogenetic approaches should deliver on all fronts; however they have yet to be widely applied.

Traditionally, the field of behavioral neuroscience has focused on examination of the behavioral effects of circumscribed and complete lesions to target structures which have not typically been restricted to particular neurotransmitter pathways. More recent work has provided clear evidence that dose-related effects of both focal neurotoxic lesions and drug treatments can be identified with dissociable behavioral effects, different from (or even contrary to) those of conventional lesions. At present we need to combine different techniques to achieve pharmacological specificity in combination with anatomical and temporal resolution (Figure 2). Pharmacogenetic approaches hold great promise but have yet to be brought to bear on functional dissociations between mPFC sub-regions. Optogenetics are also likely to become an important convergent method, to identify specific circuits with fine temporal resolution (Narayanan et al., 2012; Aston-Jones and Deisseroth, 2013). Moreover, even quite complex animal learning designs have been successfully adapted such that they can be examined using optogenetics (Steinberg et al., 2013).

Taken in isolation, the conventional lesion methods which have been a first line of approach may suggest that a particular mPFC sub-region is not necessary for a particular aspect of function. Potentially, the most striking dissociation emerging is that between AC and IL which seem to play differential roles in attentional and executive processes, respectively. However, for the most part, lesions and other interventions made at AC vs. IL placements have not been systematically compared. The ambiguity surrounding the delineation of PL function may also relate to the relatively large number of studies which have targeted this structure non-selectively (Table 1). Moreover, where any such interventions are without effect, this need not exclude a neuromodulatory role, for example mediated by a particular DA receptor sub-type. Both small differences in the placement of lesions within sub-regions and the restriction of such lesions to particular neurotransmitter pathways can result in different and even opposing patterns of behavioral outcome.

### Conflict of interest statement

The authors declare that the research was conducted in the absence of any commercial or financial relationships that could be construed as a potential conflict of interest.
